# Exosomes miR‐369‐3p Alleviates Early Brain Injury After Subarachnoid Hemorrhage by Promoting Ferroptosis of M1 Microglia via Inhibiting iNOS/GPX4 Axis

**DOI:** 10.1002/brb3.70966

**Published:** 2025-10-11

**Authors:** Jian Fang, Feiyun Qin, Pengcheng Xu, Xintong Zhao, Zihuan Zhang, Dayong Xia, Jiaqiang Liu, Jiajia Yu, Liying Hu, Yuchen Wang, Zhenbao Li, Niansheng Lai

**Affiliations:** ^1^ Department of Neurosurgery, The Translational Research Institute for Neurological Disorders of Wannan Medical College The First Affiliated Hospital of Wannan Medical College (Yijishan Hospital of Wannan Medical College) Wuhu Anhui China; ^2^ The Institutes of Brain Science Wannan Medical College Wuhu Anhui China

**Keywords:** exosomes, ferroptosis, microRNA‐369‐3p, subarachnoid hemorrhage

## Abstract

**Background:**

Ferroptosis in pathophysiological mechanisms in early brain injury after subarachnoid hemorrhage (SAH‐EBI) has been demonstrated. MicroRNAs (miRNAs) are involved in various aspects of neurological disorders. A growing number of studies suggest that intense inflammation mediated by M1 microglia after subarachnoid hemorrhage (SAH) may lead to neurological damage. According to our research and related reports, exosomal miR‐369‐3p is involved in the pathophysiological process of SAH, and miR‐369‐3p has a potentially central role in regulating inflammatory responses. Therefore, targeted delivery of miR‐369‐3p across the blood‐brain barrier (BBB) into the brain to alleviate SAH‐EBI is a promising therapeutic approach.

**Methods:**

In this study, we extracted exosomes from RBCs and then modified RVG peptide onto the exosome surface using the click chemistry principle. Finally, miR‐369‐3p mimic was loaded into the RVG peptide‐modified exosomes to form RVG‐Exo/miR‐369‐3p (RVG‐Exo/miR) by electroporation. Tail vein injection of RVG‐Exo/miR was used to achieve delivery of miR‐369‐3p into the brain of SAH mice. The effect of miR‐369‐3p on SAH‐EBI was examined by neurobehavioral scores, brain water content, Fluoro‐Jade C (FJC) staining, and Nissl staining. MDA and GSH kits were used to assess the extent of ferroptosis occurrence. Western blotting analysis, immunofluorescence staining, and qRT‐PCR were used to detect the levels of each protein, mRNA, and miRNA.

**Results:**

The exosome system (RVG‐Exo/miR) successfully delivered miR‐369‐3p to the mouse central nervous system across the blood‐brain barrierBBB. This exosomal system reduced the number of M1 microglia by enhancing their sensitivity to ferroptosis by inhibiting the expression of iNOS and GPX4. In addition, miR‐369‐3p treatment alleviated neurobehavioral disorders, brain edema, and neuronal damage after SAH‐EBI.

**Conclusions:**

RVG‐Exo/miR promotes ferroptosis in M1 microglia by inhibiting the iNOS/GPX4 axis, which may be a new and effective therapeutic strategy for treating SAH‐EBI.

## Graphical abstract

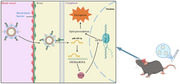

## 1 Introduction

Stroke is a significant threat to the health of the Chinese population, with subarachnoid hemorrhage accounting for approximately 1.3% of all stroke events (Tu et al. 2023). Kusaka et al. proposed early brain injury in 2004 as “early brain injury” (EBI), generally defined as within 72 h after SAH (Kusaka et al. 2004). Pathophysiological mechanisms in SAH‐EBI include neuroinflammatory response, oxidative stress, mitochondrial dysfunction, brain edema, BBB damage, and cell death (apoptosis, pyroptosis) (Lauzier et al. 2023; Fan et al. 2021; Chen et al. 2021). Microglia are resident immune cells in the central nervous system (CNS) and a mononuclear macrophage type; their activation and accumulation are considered important indicators of neuroinflammation (Coulibaly and Provencio 2020; Ransohoff and Cardona 2010; Zheng et al. 2020). In SAH‐EBI, microglia are activated, and the activated microglia polarized into two states, M1 (pro‐inflammatory) and M2 (anti‐inflammatory), and M1 microglia releasing inflammatory and chemotactic factors (IL‐1β, IL‐6, TNF‐α, iNOS, SDF‐1), which mediate neuroinflammatory responses and made neurological impairments more severe (Tao et al. 2023; Wei et al. 2021).

Ferroptosis is a newly discovered form of iron‐dependent cell death, distinct from other forms of cell necrosis, apoptosis, and autophagy (Tang et al. 2021). After subarachnoid hemorrhage, the rupture of blood vessels causes red blood cells to enter the subarachnoid space and lyse. The lysed red blood cells release a large amount of hemoglobin/heme, which are metabolized by microglia and macrophages to form ferrous/iron ions, increasing the concentration of iron ions (Deng et al. 2023; Shi et al. 2024). Microglia produce large amounts of highly toxic reactive oxygen species (ROS) through the Fenton reaction using iron as a catalyst, and they damage lipid membranes, proteins, and nucleic acids, causing irreversible cellular damage and disrupting cellular function, ultimately leading to neuronal cell death (Ge et al. 2024; Shi et al. 2024). Therefore, we found that M1 microglia assume a deleterious role in EBI in subarachnoid hemorrhage, so it is worth investigating whether the promotion of microglial iron death can be a key factor in ameliorating EBI in subarachnoid hemorrhage. However, we have limited research on the mechanisms regulating microglial iron death in SAH‐EBI.

MicroRNAs (miRNAs) are small ribonucleic acid (RNA) molecules that regulate gene expression by binding to specific messenger RNAs (mRNAs) (Singh et al. 2013; O'Connell et al. 2010; Mittelbrunn et al. 2011). In immune cells, miRNAs regulate cell proliferation, cell differentiation, and the production of inflammatory mediators (Singh et al. 2013; O'Connell et al. 2010; Mehta and Baltimore 2016). In Liu's study, miR‐369‐3p was involved in regulating autophagy in endometrioid adenocarcinoma (EEC) and inhibited endometrioid adenocarcinoma (EEC) cell proliferation by targeting autophagy‐related protein 10 (ATG10) (Liu et al. 2019). Viviana's study has demonstrated that miR‐369‐3p regulates the release of pro‐inflammatory cytokines and activation of the NF‐kB signaling pathway by modulating the expression of C/EBP‐β and iNOS (Galleggiante et al. 2019; Scalavino et al. 2020, 2023). In our previous study, 24 h after subarachnoid hemorrhage, we took plasma from patients and healthy controls. Patient peripheral blood endosomal miRNA profiling was then performed using next‐generation sequencing (NCG) and validated using quantitative real‐time PCR (qRT‐PCR). We found significant variability of miR‐369‐3p within patient exosomes (Lai et al. 2020).

Exosomes (50–150 nm in diameter) are a significant type of secreted extracellular vesicle (EV) derived from intraluminal vesicles (ILVs) in the early endosomal compartments and released from multivesicular bodies (MVBs) fused to the plasma membrane (PM) (Colombo et al. 2014; Krylova and Feng 2023). Exosomes are essential mediators that mediate intercellular communication, and they carry a variety of biomolecules from donor cells, such as miRNAs, proteins, metabolites, and lipids for transfer to recipient cells (Wortzel et al. 2019). The BBB is a highly selective biological (physiological protective) barrier; more than 98% of drugs cannot cross the BBB (Pardridge 2009; Hartl et al. 2023). Therefore, overcoming the blood‐brain barrier and fully realizing drug delivery across the BBB is a key challenge in developing drugs for treating CNS disorders. Therapeutic strategies to cross the BBB have evolved recently, such as receptor‐mediated transcytosis and neurotropic viruses, nanoparticles, and exosomes (Terstappen et al. 2021). Exosomes are well‐studied extracellular vesicles that have been used as carriers for intracranial delivery of drugs for the treatment of several CNS disorders due to their nanoscale effects, biocompatibility, long‐range targeting, circulatory stability, low immunogenicity, and crossing of the blood‐brain barrier (Khongkow et al. 2019; Zheng et al. 2019; Wang et al. 2021; Qu et al. 2018; Tian et al. 2018). The use of human red blood cells to produce exosomes for delivery of drug carriers has more favorable characteristics because (i) RBCs are the most abundant cell type in the body and are relatively easy to obtain, and they have long been routinely used for blood transfusions (Usman et al. 2018); (ii) RBCs can produce large amounts of exosomes and are not cytotoxic (Chiangjong et al. 2021); and (iii) Because RBCs lack mitochondrial and nuclear DNA, there is no risk of horizontal gene transfer (Usman et al. 2018). Using genetic engineering techniques to add Lamp2b (lysosomal‐associated membrane protein 2b)‐RVG (rabies virus glycoprotein) to the surface of exosomes, modified exosomes (RVG‐Exo) have been shown to cross the BBB and precisely deliver therapeutic nucleic acids to the central nervous system (Alvarez‐Erviti et al. 2011; Tang et al. 2023). In addition, Haroon et al. used click chemistry to design exosomes with CNS‐targeted peptide rabies virus glycoprotein (RVG29), and Yang et al. used click chemistry to design nanoplatelets with CNS‐targeted rabies virus glycoprotein peptide (RVG). They penetrate the BBB effectively and deliver the neuroprotective peptides NR2B9c and miR‐375 to the CNS (Haroon et al. 2024; Yang et al. 2024).

To our knowledge, our study is the first to treat SAH via exosomal miRNA‐mediated ferroptosis. We successfully constructed RVG‐Exo/miR in this experiment using bio‐orthogonal click chemistry and electroporation. Subsequently, experiments were performed to investigate whether RVG‐Exo/miR could deliver miR‐369‐3p to microglia and explore whether miR‐369‐3p could promote M1 microglia ferroptosis to attenuate SAH‐EBI.

## 2 Materials and Methods

### 2.1 Animals

Male C57BL/6 mice, weighing 25–30 g and aged 8–10 weeks, were purchased from Collective Pharmachem Biotechnology Co. Ltd (Jiangsu, China) and housed in the Laboratory Animal Centre of the First Affiliated Hospital of Wannan Medical College. They were housed in a suitable living environment with a 12‐h dark‐light cycle and given enough qualified feed and drinking water. The Ethics Committee of the First Affiliated Hospital of Wannan Medical College approved the study and strictly followed the National Institutes of Health guidelines for animal care and use.

### 2.2 SAH Model in Mice

This experiment was performed as previously reported with some modifications (Lai et al. 2020). Mice were anesthetized intraperitoneally (0.2 mL/10 g) with tribromoethanol (1.25% Avertin). An incision was made in the middle of the mouse scalp to expose the skull, which was fixed to the brain stereotactic apparatus. A hole was drilled on the surface of the skull 3.5 mm anterior to the bregma, and a 27‐gauge needle was slowly inserted at an inclination of 30° until it reached the base of the skull, with the needle set back 0.5 mm. Then, 0.1 mL of allogeneic nonheparinized arterial blood was injected into the anterior pool of the visual crossover for 30 s. Mice in the sham‐operated group were injected with an equal amount of saline in the anterior pool of the visual crossover. After the injection, the needle was withdrawn after a 5‐min dwell time. Finally, bone wax was used to close the puncture hole and suture the scalp. To prevent dehydration, mice were injected subcutaneously with 1 mL of 0.9% saline after surgery. We observed that the basal inferior temporal lobe was stained by blood after SAH.

### 2.3 Preparation of Exosomes

Group O blood samples were obtained from the Blood Center of Wuhu City in Anhui Province with informed consent. The following manipulations were performed as already reported in the study, with some modifications (Usman et al. 2018; Krishnan et al. 2024; Peng et al. 2023). Red blood cells were separated from plasma and leukocytes using centrifugation and a leukocyte filter. The isolated RBCs were diluted with DPBS and treated with a 10 mM calcium ion carrier (Sigma‐Aldrich, USA) overnight. To isolate exosomes, RBCs and cellular debris were removed by centrifugation at 600 × *g* for 20 min, 1600 × *g* for 15 min, 3260 × *g* for 15 min, and 10,000 × *g* for 30 min at 4°C using an ultracentrifuge (Sigma‐Aldrich, USA). The supernatant was then passed through a 0.22 µm filter. After that, the supernatant was centrifuged at 13,000 × *g* for 30 min at 4°C; after collecting the supernatant, the centrifugation was continued at 100,000 × *g* for 1 h at 4°C; the supernatant was then discarded, which was the RBC‐derived exosomes (RBC‐Exo) precipitate. Subsequently, the RBC‐Exo precipitates were resuspended with DPBS and RIPA lysate, respectively, after which they were determined using a transmission electron microscope (TEM), nanoparticle tracking analysis (NTA), and Western blotting.

### 2.4 Conjugation of RVG Peptides and Exo Surface Labeling

We first introduced the azide groups into the RVG29 peptide to form the RVG29‐Azide peptide, which GL Biochem (Shanghai) Ltd did. Then, RBC‐Exo (0.5 mg/mL) suspended in 100 µL of 1× DPBS and 100 µL of 2 µM DBCO‐PEG4‐NHS ester (No. 764019, Sigma‐Aldrich) were reacted for 5 h at room temperature. Afterward, DBCO‐Exo was obtained by washing the unreacted DBCO‐PEG4‐NHS ester molecules by centrifugation at 7500 × *g* using a Nanosep100K (Pall Life Sciences, USA) ultrafiltration column on three consecutive occasions. Subsequently, DBCO‐Exo (0.4 mg/mL), RVG29‐Azide peptide (0.6 mg/mL), and Cy5.5 (0.4 µM) were stirred at 4°C for 12 h. Finally, RVG‐Exo was again concentrated by centrifugation at 7500 × *g* in 1× DPBS using a Nanosep100K ultrafiltration column three consecutive times. RVG‐Exo (1 × 10^8^ particles/mL) were stained using 2 µM DIO (green light) lipophilic dyes and incubated for 10 min at room temperature. Unreacted dye was removed by three consecutive centrifugations (7500 × *g*) through a Nanosep100K ultrafiltration column.

### 2.5 MiR‐369‐3p Loading

Exosomes with a total protein concentration of 20 µg (detected using the BCA Assay kit, Beyotime) and 20 µL of miR‐369‐3p mimics or scrambled miRNAs (GenePharma, China) were mixed in 180 µL of nucleofector buffer (Cell Line nucleoector kit V, Amaxa) and electroporated in the Nucleofector IIs/2b device at 350 V and 150 µF. Exosomes were washed in PBS (4°C) to remove unbound miRNA by sequential ultracentrifugation. The efficiency of transfection was verified using qRT‐PCR to detect miR‐369‐3p levels.

### 2.6 RNA Isolation and qRT‐PCR

We followed the steps of RNeasy Universal Kits (Qiagen, Germany) to extract total RNA from exosomes or brain tissue. FastKing One‐Step RT‐qPCR Kit (SYBR Green, Tiangen Biotech, China) was used to analyze the mRNA level. Then, to analyze the miRNA levels, total RNA was reverse transcribed to cDNA using miRcute Plus miRNA First‐Strand cDNA Kit (Tiangen Biotech, China). Subsequently, qRT‐PCR was performed using miRcute Plus miRNA qPCR Kit (SYBR Green; Tiangen Biotech, China) for qRT‐PCR detection. All PCR reactions were performed in three replicates with mRNA or miRNA levels expressed relative to GAPDH or U6 snRNA levels. The results of PCR reactions for mRNA or miRNA expression were calculated using the 2^−△△Ct^ method. Genepharam Co. Ltd, China, synthesized all the primers. Primers used were listed as follows:

miR‐369‐3p forward: 5′‐accggccgcggAATAATACATGGTTGATCT TTT‐3′, reverse: 5′‐CAGGTCCAGTTTTTTTTTTTTTTTCGT‐3′; U6 forward: 5′‐CTCGCTTCGGCAGCACA‐3′, reverse: 5′‐AACGCTTCACGAATTTGCGT‐3′; iNOS forward: 5′‐CTCTATGTTTGCGGGGATGT‐3′, reverse: 5′‐TTCTTCGCCTCGTAAGGAAA‐3′; GAPDH forward: 5′‐ACGGCAAATTCAACGGCACAG‐3′, reverse: 5′‐ACACCAGTAGACTCCACGACATAC‐3′.

### 2.7 Western Blot Analysis

Purified exosomes or brain tissues were lysed in RIPA lysate containing protease inhibitors and then centrifuged to extract the protein supernatant. Subsequently, protein sample concentration was determined using the BCA Protein Concentration Assay Kit (Beyotime, China). Protein supernatants were denatured at 95°C for 10 min by mixing with 5× Protein loading buffer. Two‐color pre‐stained protein marker (Epizyme Biotech, Shanghai, China) and protein samples were separated by electrophoresis on 10% SDS‐PAGE (Epizyme Biotech, Shanghai, China), followed by electrophoretic transfer to a PVDF membrane (Millipore, USA). Subsequently, the PVDF membranes were closed in 5% skimmed milk for 2 h at room temperature and incubated with specific primary antibodies at 4°C overnight. Primary antibodies included: rabbit anti‐iNOS (1:1000, # ER1706‐89, HUABIO, China), rabbit anti‐CD86 (1:1000, # ET1606‐50, HUABIO, China), rabbit anti‐GPX4 (1:5000, ab125066, Abcam, USA), rabbit anti‐β‐tubulin (1:10000, # ET1602‐4, HUABIO, China), rabbit anti‐TSG101 (1:5000, ab133586, Abcam, USA), rabbit anti‐CD63 (1:5000, ab134045, Abcam, USA), rabbit anti‐Alix (1:1000, ab275377, Abcam, USA), rabbit anti‐hemoglobin α (1:1000, ab92492, Abcam, USA), rabbit anti‐GAPDH (1:10000, # ET1601‐4, HUABIO, China). The membrane was washed three times using TBST, followed by incubation and enzyme‐labeled anti‐rabbit secondary antibody (1:50,000, # HA1001 HUABIO, China) for 1 h at room temperature. Finally, the bands were visualized using an ultrasensitive ECL chemiluminescence kit (Beyotime, China) and quantified using Image J software (NIH, USA).

### 2.8 Immunofluorescence Staining

Paraffin sections of mouse brain tissue were deparaffinized, rehydrated, and subjected to antigenic repair in a modified sodium citrate antigen repair solution (Beyotime, China). Subsequently, the membranes were incubated with hydrogen peroxide for 15 min at room temperature to block endogenous peroxidase activity, followed by Triton X‐100 incubation for 15 min for membrane‐breaking, and closed with 5% BSA for 1 h. Next, incubate with primary antibodies overnight at 4°C. The primary antibodies were as follows: rabbit anti‐iNOS (1:100, # ER1706‐89, HUABIO, China), goat anti‐Iba‐1 (1:100, ab289874, Abcam, USA), rabbit anti‐Iba‐1 (1:100, **# **ET1705‐78, HUABIO, China), and rabbit anti‐GPX4 (1:100, ab125066, Abcam, USA). After that, the slides were rinsed three times with PBS and then incubated with fluorescence‐coupled secondary antibodies for 1 h at room temperature. The secondary antibodies were as follows: anti‐goat Alexa Fluor 647 (1:200, ab150131, Abcam, USA) and anti‐rabbit Alexa Fluor 488 (1:500, # HA1121, HUABIO, China). Finally, slides were blocked by the dropwise addition of DAPI‐containing anti‐fluorescence quenching blocking solution (Beyotime, China). The prepared frozen sections of mouse brain were taken, rinsed with PBS and then blocked with 5% BSA for 1 h. The rest of the steps were as above. An independent observer obtained images under a fluorescence microscope (AXIO OBSERVER 3, Zeiss, Germany).

### 2.9 Fluoro‐Jade C Staining

Fluoro‐Jade C (FJC) Ready‐to‐Dilute Staining Kit for identifying Degenerating Neurons was used to evaluate injured neurons. Paraffin sections of mouse brain tissue were deparaffinized, rehydrated, and incubated with potassium permanganate for 10 min. After that, they were incubated with FJC solution for 10 min under dark conditions and with DAPI. The slides were dried at 60°C for 5 min and then transparent in xylene for 5 min. Finally, the slides were blocked using DPX (Sigma, USA). An independent observer acquired images under a fluorescence microscope (AXIO OBSERVER 3, Zeiss, Germany). ImageJ software was used for quantitative analysis.

### 2.10 Nissl Staining

Paraffin sections of mouse brain tissue were deparaffinized and rehydrated. The sections were incubated with Nissl staining solution (Beyotime, China) for 20 min at 37°C. Compared with normal neuronal cells, post‐injury neuronal cells in vivo were seen to have shrunken and/or contained vacuoles, and the nuclei stained more deeply (Rui et al. 2021). The images were obtained under a light microscope (AXIO OBSERVER 3, Zeiss).

### 2.11 Evaluation of Ferroptosis

Ferroptosis was assessed by detecting MDA and GSH levels in brain tissues from the infratemporal cortex region of mice. According to the manufacturer's product instructions, MDA and GSH levels in mouse brain tissues were measured using a lipid peroxidation MDA assay kit (Beyotime, China) and a GSH/GSSG assay kit (Beyotime, China). The levels of MDA and GSH were determined using a microplate reader (BioTek Epoch 2, USA). MDA levels and GSH levels were expressed as µmol/mg protein.

### 2.12 Brain Edema

Brain edema was evaluated by determining brain water content (wet weight/dry weight). At 24 h post‐SAH, mice were anesthetized, and brain tissue was removed intact. Brain tissue consists of three parts: the right hemisphere, the left hemisphere, and the cerebellum. The brain samples were first weighed for wet weight (WW). The brain samples were dried at 100°C for 72 h to obtain the dry weight (DW). The water content of the brain was determined as (WW—DW)/WW × 100%.

### 2.13 Neurological Function Score

In this experiment, an independent observer used a modified Garcia score to detect neurological impairment in mice. The modified Garcia score consists of spontaneous activity (0–3 points), symmetry of all four limbs to move (0–3 points), forepaw extension (0–3 points), climbing (1–3 points), body proprioception after trunk contact (1–3 points), and response to whisker stimulation (1–3 points). The modified Garcia score is a scoring system using a scale of 3–18, with higher scores indicating better neurologic function.

### 2.14 Behavioral Tests

In this experiment, two independent observers used the balance beam test (BWT) and the forelimb placement test (FPT) to evaluate sensorimotor function.

Before the experiment, the mice were moved slowly up and down to minimize resistance movements. The mouse trunk was immobilized to ensure free movement of the forelimbs. Each side of the forelimb experiment was elicited after the ipsilateral whisker touched the corner of the table, and the number of times the ipsilateral forelimb was correctly placed on the corner of the table after the whisker was touched was recorded. Each mouse was tested 10 times on each side of the forelimb. Normal mice could rapidly place the ipsilateral forelimb to the corner of the table after whisker touching, and injured mice showed a delayed response.

The balance beam test (BWT) is rated on a scale of 0–5: 40 s without any movement and falling off the beam (0 points), no movement for 40 s but no fall off the beam (1 point), moving no more than half the length of the beam in 40 s (2 points), moving more than half the length of the beam in 40 s (3 points), reaching any platform at both ends within 40 s (4 points), reaching any platform at both ends in 25 s (5 points). Higher scores indicate better motor integration and coordination of the mice.

### 2.15 Statistical Analysis

Data were analyzed using GraphPad Prism 8 software, and graphs were generated. All experiments were repeated at least three times. All quantitative data are expressed as mean ± standard deviation (mean ± SD). The Mann‐Whitney U or Student's *t*‐test (two‐tailed, unpaired) was only used to compare the two experimental groups. For comparisons of more than two groups, statistical analyses were performed using analysis of variance (one‐way or two‐way) followed by Tukey's post hoc test. In all statistical analyses, *p* values were two‐sided, and *p* < 0.05 was considered significant.

## 3 Results

### 3.1 Preparation and Characterization of RBC‐Derived Exo

**FIGURE 1:**
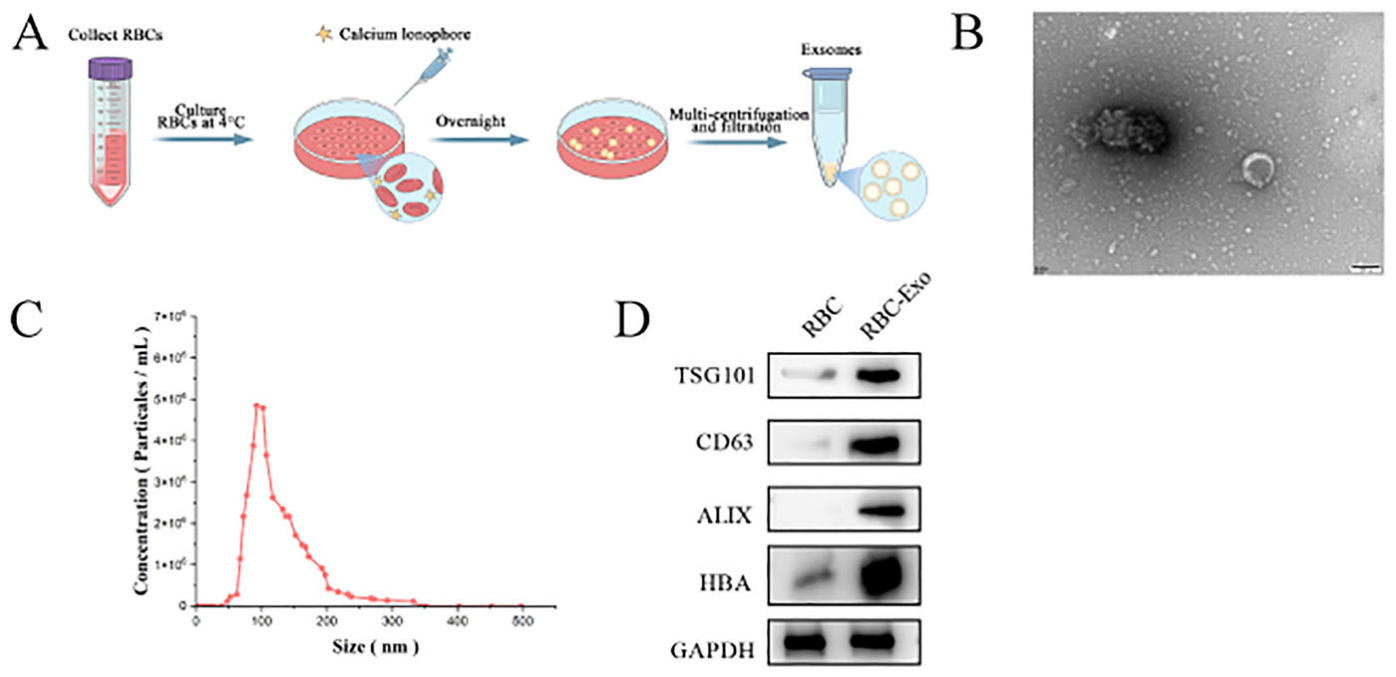
Characterization of exosomes from RBCs. (A) Stepwise graph of extraction, differential centrifugation, and purification of RBC‐Exo. (B) Transmission electron micrograph of RBC‐Exo. Scale bar: 100 nm. (C) Measurement of the particle size distribution of RBC‐Exo using the NTA method. (D) Western blot analysis of erythrocyte marker hemoglobin A (HBA) and exosome markers TSG101, Alix, and CD63 isolated from RBC‐Exo and cell lysates.

We extracted large numbers of red blood cells from blood samples from healthy donors. RBCs were treated with calcium ionophore overnight to increase the release of exosomes from the RBCs. Then, RBC‐derived Exo (RBC‐Exo) was isolated and purified by differential centrifugation and filtration treatment (Figure 1A). RBC‐Exo morphology was observed using TEM, which showed that RBC‐Exo presented a hemispherical shape with one side concave (Figure 1B), consistent with the unique microstructure of exosomes. RBC‐Exo was examined for particle size by NTA, which showed that RBC‐Exo particle size ranges from approximately 114.4 ± 59.3 nm (Figure 1C). Afterward, Western blot was used to detect exosome‐specific protein markers (Alix, CD63, TSG101) and the major RBC protein hemoglobin A (HBA). Figure 1D shows that RBC‐Exo is enriched in exosome‐positive marker proteins and hemoglobin A. Combined with the analysis above, it shows that exosomes of RBC origin are consistent with the characteristics of exosomes.

### 3.2 Synthesis and Characterization of RVG‐Exo/miR‐Targeted Delivery System for the CNS

**FIGURE 2:**
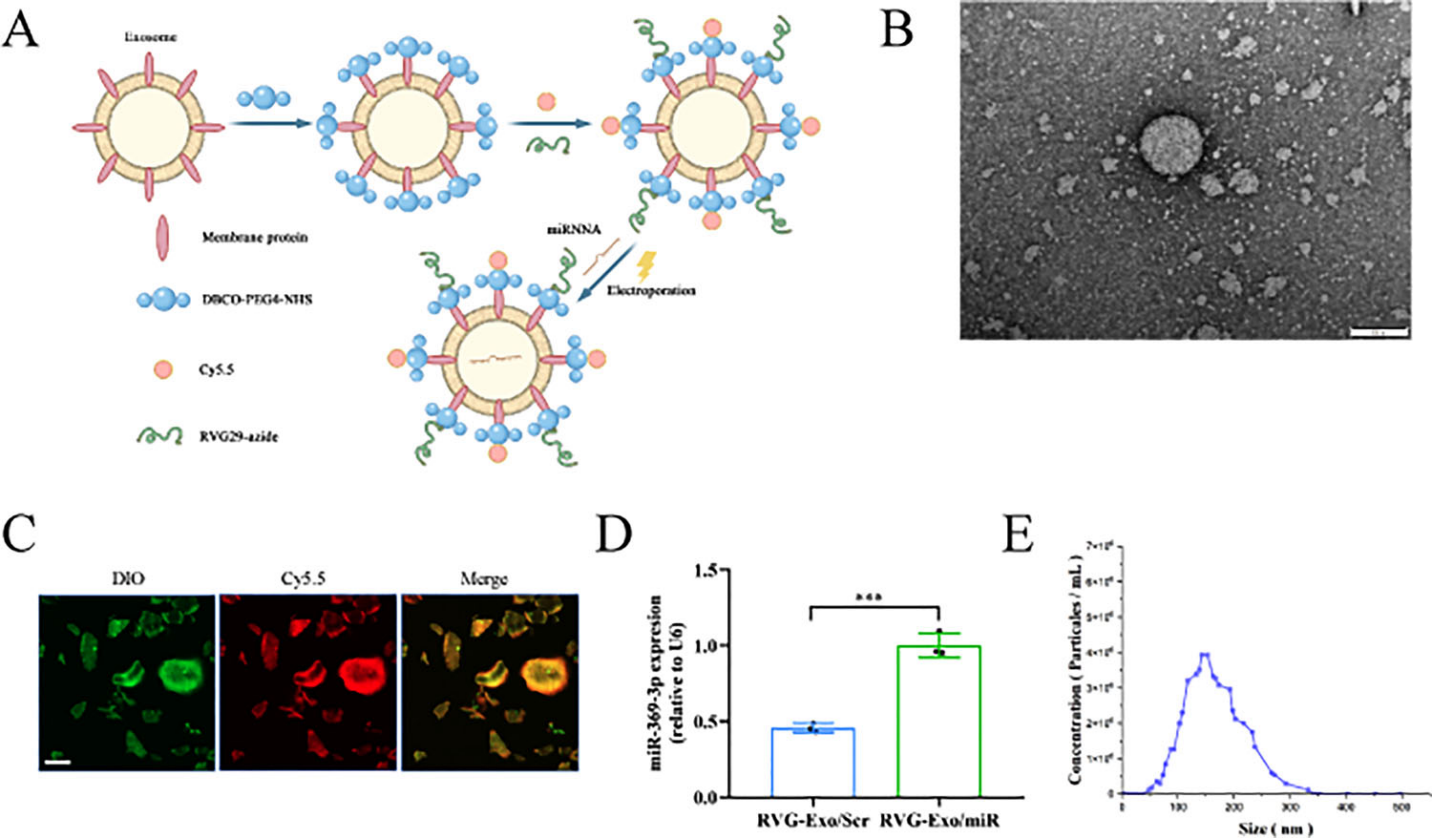
Synthesis and characterization of the RVG‐Exo/miR delivery system. (A) Schematic picture of the formation of RVG‐Exo/miR by the click chemistry reaction principle and electroporation method. (B) Transmission electron micrograph of RVG‐Exo. Scale bar: 100 nm. (C) Measurement of the particle size distribution of RVG‐Exo using the NTA method. (D) Images of DIO‐labeled RBC‐Exo and Cy5.5‐labeled proteins successfully coupled by click chemistry. Scale bar: 20 µm. (E) Expression levels of miR‐369‐3p in RVG‐Exo/scrambled miRNAs (control) and RVR‐Exo/miR‐369‐3p were analyzed using qRT‐PCR. Data were normalized to U6 expression and expressed as mean ± SD (**p < 0.01).

Subsequently, we specifically targeted the central nervous system (CNS) by designing and synthesizing RVG‐Exo/miR (Figure 2A).

First, DBCO‐PEG4‐NHS was used to cross‐link with RBC‐Exo to modify the DBCO groups onto the surface of RBC‐Exo. Then, RVG29‐Azide and DBCO‐Exo were coupled by copper‐free click chemistry to form RVG‐Exo. Copper‐free click chemistry is a simple and efficient linkage performed in water, in a buffer, and under physiological conditions without catalysts. It modifies exosomes according to their needs. The morphology of RVG‐Exo was observed using TEM, and RVG‐Exo still showed a hemispherical shape with one side concave (Figure 2B). The particle size distribution of the RVG‐Exo samples was also analyzed, and the results showed that the particle size of the RVG‐Exo samples was 169.3 ± 57.5 nm (Figure 2C). Combined analysis of Figure 2B,C showed that the particle size of the RVG‐Exo sample increased from 114.4 ± 59.3 nm in the RBC‐Exo sample to 169.3 ± 57.5 nm, which may be formed by the successful binding of the RVG moiety to the exosome surface. To visualize RVG‐Exo, Cy5.5 (red light) was also coupled to the DBCO moiety, followed by labeling the exosome membrane with DIO (green). As shown in Figure 2D, DIO and Cy5.5 co‐localized fluorescence images identified successful click chemistry on the exosome surface. Finally, miR‐369‐3p mimics or disordered miRNAs were loaded into RVG‐Exo to form a targeted CNS exosome system (named RVG‐Exo/miR and RVG‐Exo/Scr, respectively) by using electroporation. The levels of miR‐369‐3p in the exosome systems (RVG‐Exo/miR, RVG‐Exo/Scr) were analyzed using qRT‐PCR. The results showed that miR‐369‐3p levels were significantly higher in RVG‐Exo/miR than in RVG‐Exo/Scr (Figure 2E).

### 3.3 Determination of the Targeting Capability of RVG‐Exo/miR in the Brain

**FIGURE 3:**
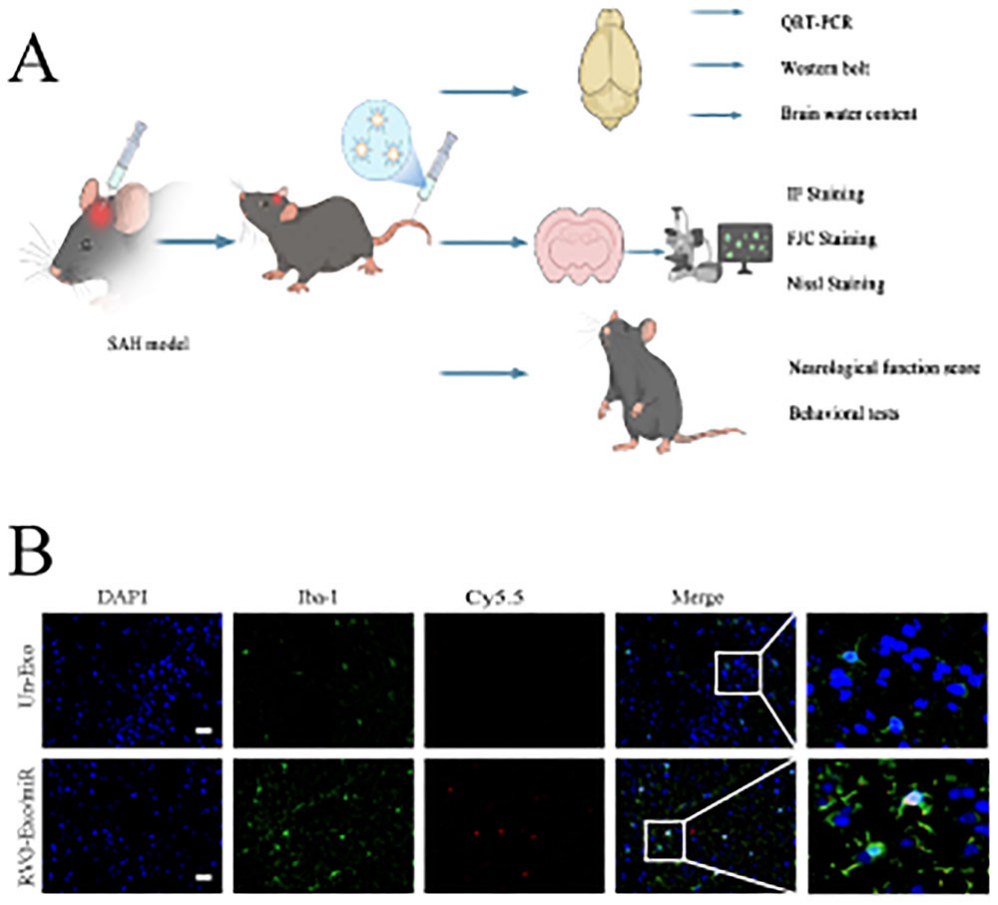
Framework for experimental design and increased exosome targeting in the brain after subarachnoid hemorrhage in mice. (A) The flow chart of a series of in vivo experiments performed after constructing the SAH mouse model. (B) Immunofluorescence images of mouse brain tissue after SAH, where RVR‐Exo/miR was successfully targeted to the CNS and labeled in microglia. The right images (last column only) represent magnified images from the boxes in the left column. Scale bar: 20 µm.

**FIGURE 4:**
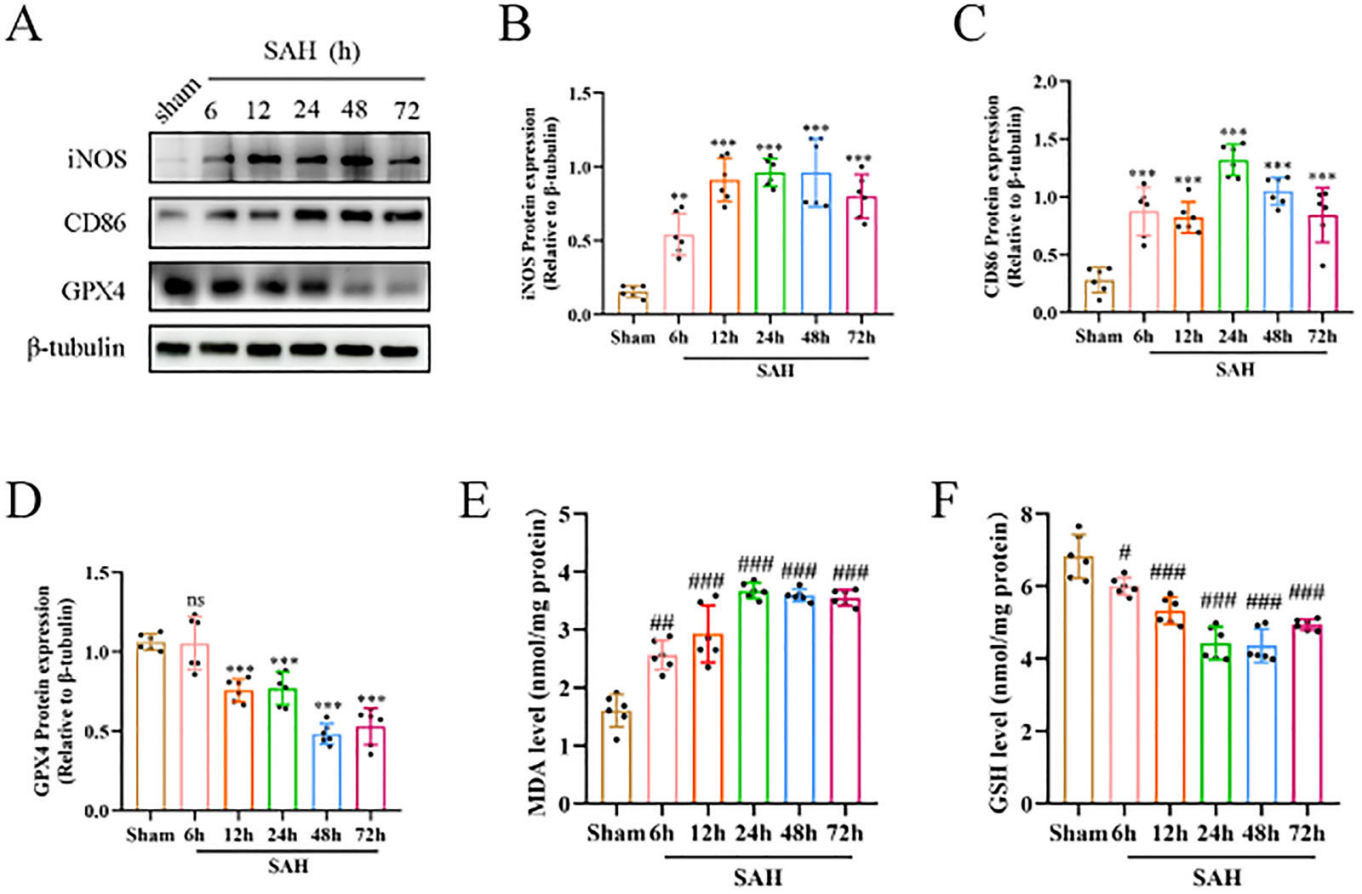
To observe changes in iNOS, CD86, and GPX4 and to explore trends in ferroptosis in a mouse model of SAH. (A–D) Protein immunoblotting and quantitative analyses of iNOS, CD86, and GPX4 in mice in the Sham group and the 6, 12, 24, 48, and 72 h groups after SAH (*n* = 6). (E) Quantitative analysis of MDA levels in the mouse SAH model (*n* = 6). (F) Quantitative analysis of GSH levels in the mouse SAH model (*n* = 6). (**p < 0.01, ***p < 0.001, #*p* < 0.05, ##*p* < 0.01, ####*p* < 0.001, ns, not significant).

We injected unmodified RBC‐Exo (Un‐Exo) and RVG‐Exo/miR via the tail vein 24 h after SAH, deeply anesthetized the mice 2 h later, and immediately removed the brain tissues for subsequent IF staining experiments (step‐by‐step Figure 3A). IF staining for Iba‐1, a biomarker for microglia, was performed, along with co‐staining of microglia nuclei with DAPI. Cy5.5‐labeled RVG‐Exo/miR was found to co‐localize with microglia, suggesting that intravenously injected RVG‐Exo/miR could be taken up by microglia (as shown in Figure 1G). Incidentally, the intensity and distribution of Cy5.5 (red) in RVG‐Exo/miR were significantly higher than that of Un‐Exo (Figure 4A), which demonstrated that RVG peptide‐modified exosomes could dramatically enhance the ability of the exosome carrier delivery system to target the CNS specifically.

### 3.4 M1 Microglia Are Activated, and Ferroptosis Occurs in the Brain After SAH

After the SAH mouse model was established, we collected brain tissues for experiments using 6, 12, 24, 48, and 72 h time points during the SAH‐EBI period. To observe the expression of M1 microglia and ferroptosis within SAH‐EBI, we detected M1 microglia‐specific markers (iNOS and CD86) and GPX4, a core regulator of ferroptosis (Tang et al. 2021; Qu et al. 2022). Protein expression levels of CD86, iNOS, and GPX4 in the temporal cortex of mice after SAH were measured by Western blot at 6, 12, 24, 48, and 72 h. As shown in the results of Figure 4A–D, the protein levels of CD86 and iNOS were significantly elevated after SAH compared to the Sham group, in contrast to the reduced expression of protein levels of GPX4. These results demonstrate that microglia within SAH‐EBI are polarized toward M1 microglia. It has been largely demonstrated that reduced levels of GPX4 expression following the occurrence of ferroptosis (Shi et al. 2024; Gaschler et al. 2018). Following, we examined two additional ferroptosis biomarkers, including glutathione (GSH), an essential substrate of GPX4, and lipid peroxidation‐derived malondialdehyde (MDA). We found that GSH levels were reduced during SAH‐EBI, and MDA content was upregulated compared to the Sham group (Figure 4E,F). Since reduced GSH levels can cause ferroptosis, and MDA is the final product of ferroptosis (Rui et al. 2021), SAH‐EBI ferroptosis occurs when analyzed with the above results.

### 3.5 RVG‐Exo/miR Promotes Ferroptosis in M1 Microglia by Inhibiting the iNOS/GPX4 Axis

**FIGURE 5:**
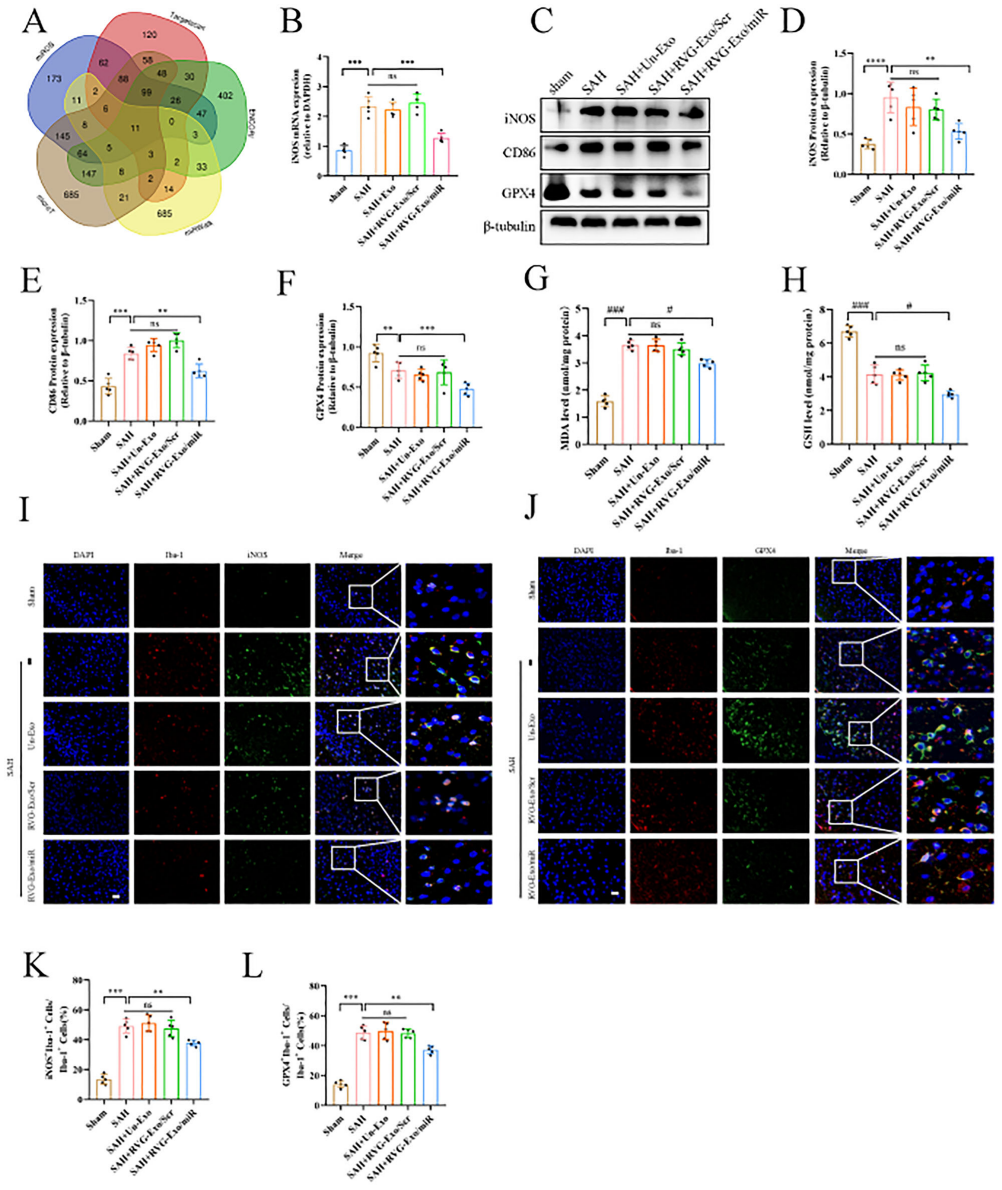
RVG‐Exo/miR promotes ferroptosis in M1 microglia after SAH by inhibiting the iNOS/GPX4 axis. (A, B) Bioinformatics and changes in iNOS mRNA expression were detected after SAH in vivo by qRT‐PCR after RVG‐Exo/miR treatment. Data were normalized to GAPDH expression and expressed as mean ± SD (*n* = 5). (C–F) Western blot and quantitative analysis of iNOS, CD86, and GPX4 in Sham, SAH, SAH+Un‐Exo, SAH+RVG‐Exo/Scr, and SAH+RVG‐Exo/miR groups of mice (*n* = 5). (G) Quantitative analysis of MDA levels after SAH in vivo in the Sham, SAH, SAH+Un‐Exo, SAH+RVG‐Exo/Scr, and SAH+RVG‐Exo/miR groups (*n* = 5). (H) Quantitative analysis of GSH levels after SAH in vivo in the Sham, SAH, SAH+Un‐Exo, SAH+RVG‐Exo/Scr, and SAH+RVG‐Exo/miR groups (*n* = 5). (I–L) IF staining and quantitative analysis of iNOS and GPX4 in the cortex 24 h after SAH (*n* = 5). Scale bar: 20 µm. The right images (last column only) represent magnified images from the boxes in the left column. (*p < 0.05, **p < 0.01, ***p < 0.001, #*p* < 0.05, ####*p* < 0.001; ns, not significant).

Previous studies demonstrated that the gene target of miR‐369‐3p is iNOS and that miR‐369‐3p significantly reduces intracellular iNOS mRNA and iNOS protein production (Scalavino et al. 2020). We validated the target of miR‐369‐3p by five different algorithms (Figure 5A). iNOS enhances the resistance of M1 microglia to ferroptosis, and iNOS and GPX4 play parallel roles in preventing ferroptosis after SAH (Qu et al. 2022; Kapralov et al. 2020). To explore the effect of RVG‐Exo/miR on iNOS in M1 microglia, we measured the expression of iNOS mRNA in the brain and found that the level of iNOS mRNA was reduced after RVG‐Exo/miR treatment (Figure 5B). We examined the M1 microglia‐specific markers iNOS and CD86 protein expression to validate this phenomenon. We found a significant increase in iNOS and CD86 protein expression after SAH and a moderate decrease in iNOS and CD86 expression after RVG‐Exo/miR therapy (Figure 5C–E). Then, we performed immunofluorescence staining of brain tissues to detect the specific expression of iNOS in M1 microglia. We found that iNOS expression was significantly elevated in the SAH group compared with the Sham group. Still, when RVG‐Exo/miR treatment was performed, iNOS expression in M1 microglia was significantly reduced (Figure 5I,J). These findings suggest that RVG‐Exo/miR treatment after SAH reduces M1 microglia numbers. To determine how ferroptosis affects M1 microglia. First, we assayed MDA and GSH levels to assess ferroptosis. In the MDA assay, MDA levels were more prominent in the SAH group relative to the Sham group but were moderately reduced when treated with RVG‐Exo/miR (Figure 5G). In addition, by measuring GSH, we also found that GSH levels were lower after RVG‐Exo/miR treatment than in the Sham and SAH groups (Figure 5H). To further explore whether RVG‐Exo/miR promotes ferroptosis in M1 microglia by regulating the ferroptosis mechanism. We measured the protein expression of GPX4, a core regulator of ferroptosis. We found that the protein expression was moderately reduced after SAH, and the expression level was significantly reduced after RVG‐Exo/miR treatment (Figure 5C,F). Meanwhile, we observed GPX4 expression in M1 microglia by IF staining of brain coronal sections. GPX4 expression was significantly higher in the SAH group than in the Sham group. Still, when RVG‐Exo/miR treatment was administered, the expression of GPX4 was significantly reduced in the M1 microglia (Figure 5K,L). These results indicated that ferroptosis in M1 microglia was significantly increased after RVG‐Exo/miR treatment after SAH. Therefore, according to the above results, RVG‐Exo/miR can promote M1 microglia ferroptosis by inhibiting the expression of the iNOS/GPX4 axis.

### 3.6 RVG‐Exo/miR Promotion of Ferroptosis in M1 Microglia Reduces Neuronal Cell Injury and Attenuates Brain Edema

**FIGURE 6:**
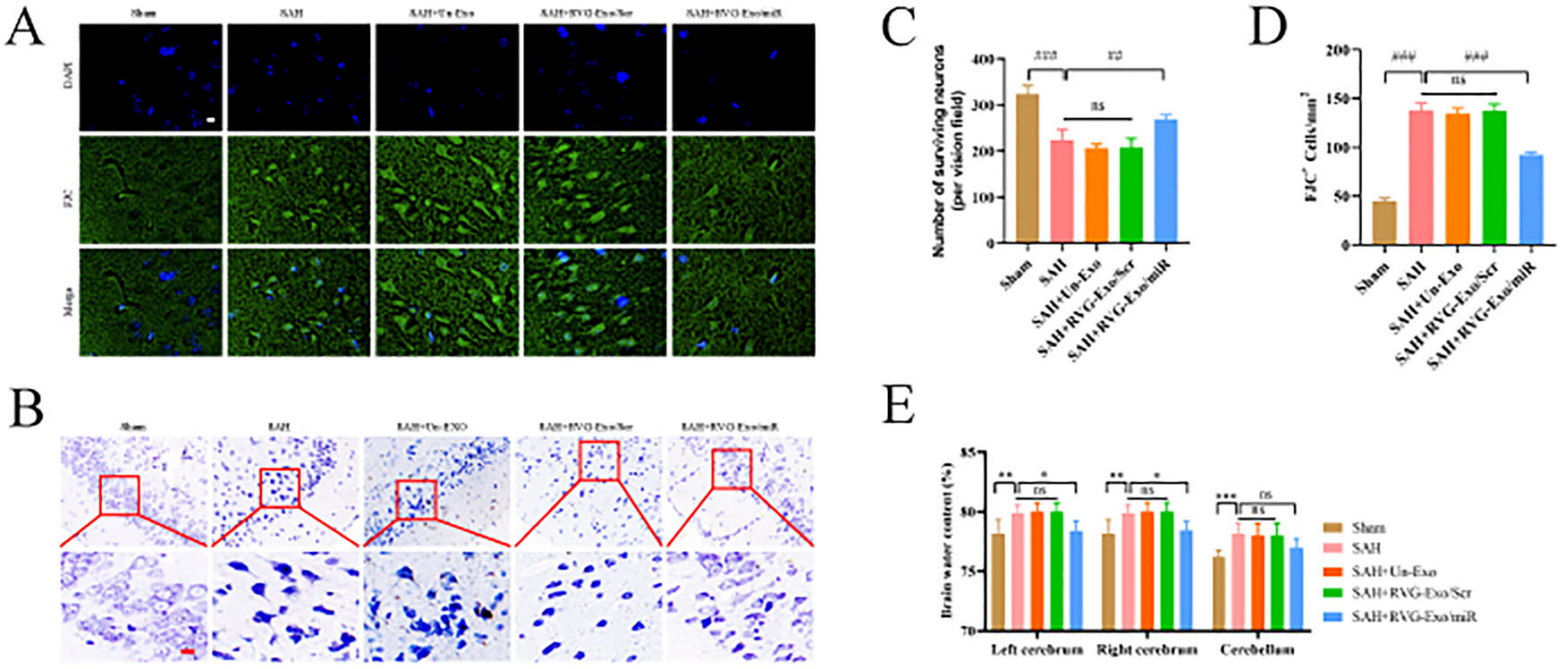
RVG‐Exo/miR reduced neuronal cell injury and alleviated brain edema through M1 microglia ferroptosis. (A, B) Changes in the number of FJC‐positive cells in FJC staining in the cortical region of the brain of mice in the Sham, SAH, SAH+Un‐Exo, SAH+RVG‐Exo/Scr, and SAH+RVG‐Exo/miR groups, and quantitative analysis (*n* = 5). (C, D) Damage to neuronal cells in cortical regions was assessed by Nissl staining (*n* = 5). (E) The wet‐DW method assessed brain water content in each group (*n* = 5). ##*p* < 0.01, ###*p* < 0.001, *p < 0.05, **p < 0.01, ***p < 0.001; ns, not significant. Scale bar: 20 µm.

To investigate the effect of miR‐369‐3p on neuronal damage after promoting iron death of M1 microglia in SAH‐EBI, we used Nissl staining and FJC staining to detect the amount of neuronal damage in the brain after SAH. According to the results of Nissl staining and FJC staining, the amount of neuronal damage in the inferotemporal cortex of mice was significantly increased after SAH was induced. At the same time, miR‐369‐3p treatment significantly increased the number of surviving neurons (Figure 6A–D). Later, we further investigated the effect of miR‐369‐3p on the degree of early brain swelling after SAH. The brain water content of different anatomical tissue structures showed that the right hemisphere, left hemisphere, and cerebellar brain water content were significantly increased in mice after SAH compared with the Sham group (Figure 6E). Critically, after treatment with miR‐369‐3p, the right hemisphere and left hemisphere brain water contents of SAH mice were decreased, while the cerebellar brain water content was almost unaffected (Figure 6E). Based on these results, miR‐369‐3p treatment attenuated neuronal injury and brain edema after subarachnoid hemorrhage.

### 3.7 RVG‐Exo/miR Promotion of Ferroptosis in M1 Microglia Alleviates Neurobehavioral Dysfunction After SAH

**FIGURE 7:**
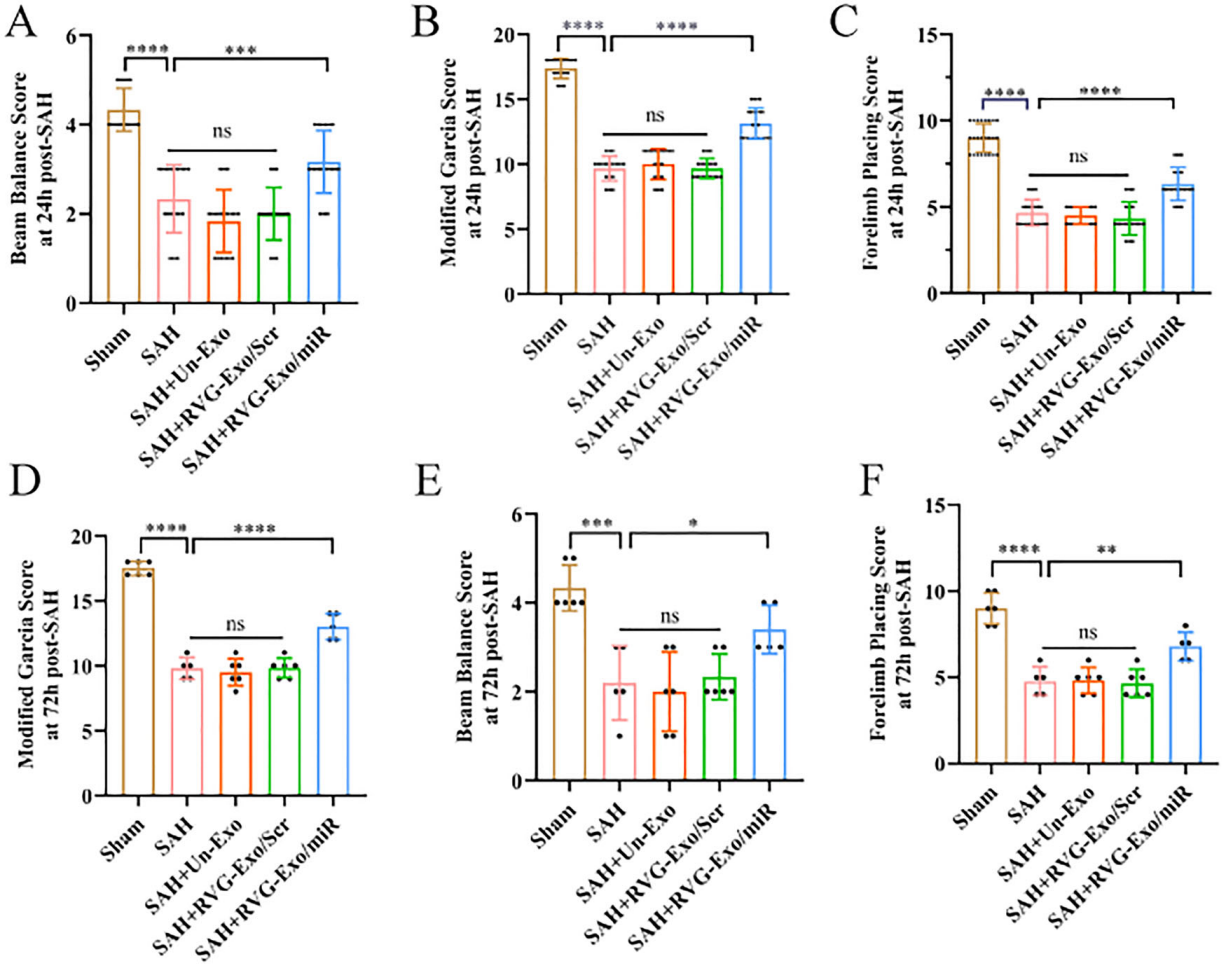
RVG‐Exo/miR mediates ferroptosis in M1 microglia to improve neurological function. (A, B) Neurologic scores were obtained 24 and 72 h after SAH using the modified Garcia scale. (C, D) Balance beam test scores were obtained 24 and 72 h after SAH. (E, F) FPT results were obtained 24 and 72 h after SAH. *n*: Look at the black spot in the picture, *p < 0.01, **p < 0.001, ***p < 0.001.

To evaluate the therapeutic effect of RVG‐Exo/miR on short‐term neurological injury after SAH, we used a modified Garcia scoring system, the Balance beam test (BWT), and the FPT. First, neurologic deficits were significantly induced after SAH compared with the Sham group, and neurologic scores were significantly reduced after receiving RVG‐Exo/miR treatment (Figure 7A,D). Then, the BWT and FPT were used to assess the motor‐sensory functions of mice after SAH, respectively. In the BWT, mice's motor coordination and balance abilities after SAH were significantly impaired compared to the Sham group (Figure 7B,E). After RVG‐Exo/miR treatment, mice improved motor coordination and balance (Figure 7B,E). Meanwhile, in the FPT, we observed that the motor‐sensory function was severely impaired in the post‐SAH mice compared with the Sham group. In contrast, the performance of the post‐SAH animals was significantly improved after RVG‐Exo/miR treatment (Figure 7C,F). In summary, SAH was able to substantially induce neurobehavioral impairment in mice, while RVG‐Exo/miR could significantly improve neurobehavioral function.

**FIGURE 8:**
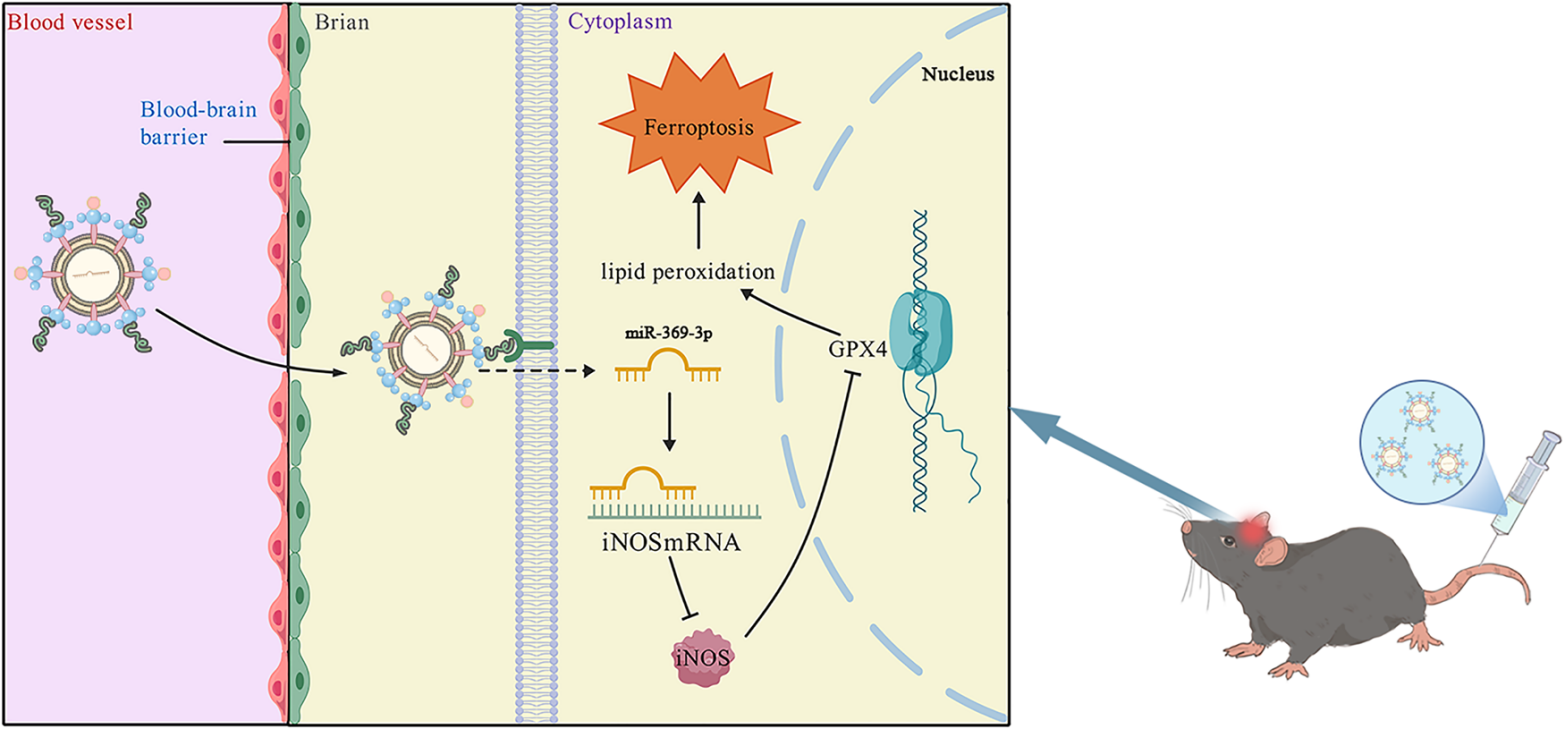
Schematic representation of RVG‐Exo/miR promoting ferroptosis in M1 microglia after subarachnoid hemorrhage by inhibiting the iNOS/GPX4 axis. After treatment with RVG‐Exo/miR via intravenous injection following subarachnoid hemorrhage, miR‐369‐3p binds to iNOS and downregulates the downstream gene GPX4, promoting ferroptosis in M1 microglia.

## 4 Discussion

SAH is a fatal acute subtype of stroke. Due to the early age of onset of SAH, high mortality and disability rates, and poor prognosis, it imposes a reduced quality of life and a severe economic burden on patients (Chen et al. 2014). The combination of early brain damage (within 72 h) and delayed brain damage after SAH contributes to this. The pathophysiologic mechanisms and processes of SAH‐EBI are complex and are associated with increased intracranial pressure, decreased cerebral blood flow, neuroinflammation, and cell death (Tao et al. 2023). The toxic cascade response induced by SAH‐EBI and the neurotoxic effects of hemoglobin and its breakdown products in the subarachnoid space are thought to be key factors in the development of delayed brain damage (Bulters et al. 2018). Therefore, the intervention of SAH‐EBI is a key point in the treatment of brain injury caused by SAH. However, the results of the current extensive studies on the complex molecular mechanisms involved in SAH‐EBI, focusing on clinical drug research and development by intervening in apoptosis, autophagy, and cerebral vasospasm, are unremarkable (Chen et al. 2024). Many studies have demonstrated that ferroptosis appears to be a viable potential target for treating SAH‐EBI.

In the current study, we used an experimental mouse model to validate the mechanism associated with the involvement of RVG‐Exo/miR in ferroptosis of M1 microglia in SAH‐EBI. We demonstrated that the iNOS/GPX4 axis is a critical factor. We also explored the role of RVG‐Exo/miR in attenuating neuronal injury, brain edema, and neurological deficits. First, we modified RBC‐Exo with RVG29 peptide targeting the CNS by click chemistry. Then, miR‐369‐3p mimic was loaded into RVG‐Exo by electroporation. Finally, we successfully constructed the engineered exosome (RVG‐Exo/miR). When applied in vivo to experimental animals, RVG‐Exo/miR demonstrated good targeting of the CNS. Second, our results indicate that in the in vivo model, the expression of the M1 microglia‐specific biomarkers iNOS and CD86 was significantly increased during SAH‐EBI. Similarly, the expression level of the anti‐ferroptosis molecule GPX4 was reduced, and the levels of the ferroptosis indicators GSH and MDA changed accordingly. Third, we observed that inhibition of the iNOS/GPX4 axis using RVG‐Exo/miR into the mouse brain after SAH exacerbated M1 microglia ferroptosis while attenuating neuronal damage. Finally, treatment with RVG‐Exo/miR ameliorated brain edema and neurobehavioral impairment. These results suggest that miR‐369‐3p is critical for controlling ferroptosis and that miR‐369‐3p may be an effective treatment for mitigating SAH‐EBI Figure 8.

In our previous study, we used NGS for plasma total exosome miRNA analysis in SAH patients and healthy controls to look for and analyze the differential expression levels of exosome miRNAs after SAH (Lai et al. 2020). We found significant differential expression of hsa‐miR‐193b‐3p and hsa‐miR‐369‐3p in total exosome miRNAs. Subsequently, in an in vivo model of SAH, we found that delivery of miR‐193b‐3p to the brain using RVG‐modified exosomes attenuated neuroinflammation after SAH (Lai et al. 2020). Therefore, we sought to explore the mechanism of miR‐369‐3p in SAH. Interestingly, in a recent study, miR‐369‐3p was able to target iNOS to reduce its expression level (Scalavino et al. 2020). After SAH, inducible nitric oxide synthase (iNOS/NOS2) is a marker and one of the essential pro‐inflammatory factors in M1 microglia (Qu et al. 2022). The presence of the BBB is a significant challenge in achieving drug delivery into the brain. Exosomes can deliver a range of biological information and cross the BBB (Wortzel et al. 2019). However, exosomes lack the targeting ability to achieve targeted delivery to the site of injury, which would significantly reduce the therapeutic effect. In this study, we constructed engineered exosomes (RVG‐Exo/miR) using electroporation and click chemistry. In an in vivo model of SAH using tail vein injection of RVG‐Exo/miR, the results showed that the modified exosome could deliver miR‐369‐3p into the brain and target binding to microglia. This demonstrates a good ability to cross the BBB and could be a promising delivery platform in the future. Meanwhile, we observed that the expression of iNOS mRNA in the brain was reduced after treatment with miR‐369‐3p. Therefore, we sought to discover the potential role of miR‐369‐3p in M1 microglia and SAH‐EBI.

After SAH, microglia can activate into either M1 (pro‐inflammatory) or M2 (anti‐inflammatory) polarized phenotype. M1 microglia can release inflammatory cytokines such as IL‐1b, TNF‐a, IL‐6, and iNOS, exacerbating neural tissue damage (Tian et al. 2022). On the one hand, iNOS is an inducible NOS isoform whose activity is controlled only by the degradation of the enzyme itself; on the other hand, microglia induced by inflammation express iNOS regardless of the in vivo and in vitro models, whereas neuronal cells do not trigger expression (Béchade et al. 2014). According to Liu's study, inhibition of iNOS using an iNOS inhibitor (14,000 W) and iNOS knockout mice had a significant protective effect on vasospasm, microvascular thrombosis, and neurologic function induced after SAH (Liu et al. 2023). Another study also showed that aminoguanidine (AG) significantly reversed cerebral vasospasm after SAH by inhibiting iNOS activity (Zheng et al. 2010). This evidence suggests that inhibition of iNOS expression plays a protective role in the mechanisms underlying the onset and progression of SAH‐induced brain injury. Likewise, our inhibition of iNOS using RVG‐Exo/miR significantly improved neuronal cell injury, brain edema, and neurological deficits induced after SAH. However, the exact molecular mechanisms in iNOS regulation of SAH‐EBI have not been effectively elucidated. In this study, we found that the expression of iNOS and CD86 was increased after SAH compared with the Sham group. These results suggest that microglia are activated into M1 microglia in large numbers after subarachnoid hemorrhage, causing increased brain damage. Kapralov's study found that iNOS may be an effective regulator of ferroptosis, acting upstream of GPX4, thereby enhancing the ability to control ferroptosis (Kapralov et al. 2020). Knocking down iNOS in M1 microglia and inducing ferroptosis with RSL3 caused an increase in the sensitivity of microglia to ferroptosis, and Fer‐1 could effectively inhibit RSL3‐induced ferroptosis.

To determine that the anti‐ferroptosis effect of iNOS was not RSL3‐unique, ML 162, an inhibitor of GPX4, and two reagents that affect the GPX4/GSH system, Imidazole ketone erastin and erastin, were used. The results found that iNOS could still downregulate the sensitivity to ferroptosis. In addition, they demonstrated that TFR, another essential regulator of ferroptosis, did not play a role in the effect of iNOS on microglia ferroptosis. Further studies on the knockdown of GPX4‐induced ferroptosis in iNOS‐enriched M1 macrophages found that ferroptosis sensitivity remained low. Meanwhile, Qu's study confirmed through a series of experiments that targeted inhibition of iNOS promotes ferroptosis in M1 microglia (Qu et al. 2022). In subsequent molecular mechanism experiments, the protein expression level of GPX4 was further reduced after iNOS inhibition in an in vitro model of SAH.

In summary, our study was conducted on the basis that iNOS and GPX4 are synergistic in controlling cellular ferroptosis and that iNOS induces ferroptosis by indirectly regulating GPX4 inactivation. Our experiments revealed that GPX4 protein levels decreased after miR‐369‐3p inhibited iNOS expression. Subsequently, the levels of MDA and GSH were examined to assess the extent of ferroptosis, which was moderately upregulated.

Ferroptosis is an iron‐dependent mode of programmed cell death that, unlike other forms of cell death, is characterized by the failure of the core mechanism of GSH/GPX4‐dependent lipid peroxidation (Tang et al. 2021). Deprivation in cysteine levels may lead to GSH depletion, which in turn reduces GPX4 levels and activity, accumulating unrepaired lipid peroxidation and lipid peroxides and ferroptotic cell death (Tang et al. 2021; Gaschler et al. 2018). After SAH, hemoglobin in the blood is metabolized by macrophages to produce large amounts of iron ions, and activated microglia in a ferrous ion‐rich environment mediate the Fenton reaction to produce large quantities of ROS, and then excess ROS production destroys intracellular nucleic acids, proteins, and cellular membranes, which further aggravates brain damage (Ge et al. 2024). In summary, we performed in vivo experiments to assess whether miR‐369‐3p promotes ferroptosis in M1 microglia. After SAH, the expression of iNOS and CD86 was upregulated, whereas the expression of GPX4 was decreased in the mouse brain. These results indicated that the number of M1 microglia increased quickly after SAH.

Meanwhile, iNOS release from M1 microglia was able to inhibit ferroptosis moderately. Notably, after we treated with miR‐369‐3p, the expression of iNOS CD86 decreased, while the expression of GPX4 was further downregulated. Meanwhile, we assayed GSH levels and detected MDA to assess lipid peroxidation. We observed that miR‐369‐3p promoted GSH depletion and lipid peroxidation. IF staining showed that the expression of iNOS and GPX4 decreased in M1 microglia after we treated with miR‐369‐3p. The above experimental results showed that the number of M1 microglia decreased after RVG‐Exo/miR treatment. Our results demonstrated that RVG‐Exo/miR could regulate and promote M1 microglia iron death after subarachnoid hemorrhage by inhibiting the iNOS/GPX4 axis. However, the mechanism of microglia ferroptosis has not yet been clarified. Although RVG‐Exo/miR inhibition of the iNOS/GPX4 axis can regulate M1 microglia ferroptosis in the brain of SAH mice, it is not the only regulatory mechanism. It may be affected by other signaling pathways.

In conclusion, we successfully constructed and validated RVG‐Exo/miRs loaded with miR‐369‐3p that can target the CNS through the BBB and the potential mechanism by which miR‐369‐3p attenuates brain injury after SAH using electroporation and click chemistry reactions. Considering the critical role of ferroptosis within SAH, these results may provide a new miRNA‐based therapeutic approach for patients with SAH. They may become an innovative treatment for SAH by promoting ferroptosis in M1 microglia. Despite this study's value for treating SAH‐induced brain injury, translation of the experimental results and clinical applications need to be followed up with further studies.

## Author Contributions

**Jian Fang**: conceptualization, methodology, validation, investigation, writing – original draft. **Feiyun Qin**: methodology, validation. **Pengcheng Xu**: methodology, validation. **Xintong Zhao**: methodology, validation. **Zihuan Zhang**: supervision, project administration, validation. **Dayong Xia**: supervision, investigation. **Jiaqiang Liu**: supervision, project administration. **Jiajia Yu**: supervision, validation. **Liying Hu**: validation, project administration. **Yuchen Wang**: supervision, project administration. **Zhenbao Li**: conceptualization, methodology, writing – review and editing, supervision, funding acquisition. **Niansheng Lai**: conceptualization, methodology, investigation, writing – original draft, supervision, project administration, funding acquisition.

## Ethics Statement

This study was approved by the Animal Care and Use Committee of Wannan Medical College (LLSC‐2023‐112) and complied with the NIH guidelines for the handling of laboratory animals.

## Conflicts of Interest

The authors declare that they have no known competing financial interests or personal relationships that could have appeared to influence the work reported in this paper.

## Peer Review

The peer review history for this article is available at https://publons.com/publon/10.1002/brb3.70966

## Data Availability

The datasets used and/or analyzed during the current study are available from the corresponding author on reasonable request.

## References

[brb370966-bib-0001] Alvarez‐Erviti, L. , Y. Seow , H. Yin , C. Betts , S. Lakhal , and M. J. Wood . 2011. “Delivery of siRNA to the Mouse Brain by Systemic Injection of Targeted Exosomes.” Nature Biotechnology 29, no. 4: 341–345. 10.1038/nbt.1807 21423189

[brb370966-bib-0002] Béchade, C. , S. Colasse , M. A. Diana , M. Rouault , and A. Bessis . 2014. “NOS2 Expression Is Restricted to Neurons in the Healthy Brain but Is Triggered in Microglia Upon Inflammation.” Glia 62, no. 6: 956–963. 24615726 10.1002/glia.22652

[brb370966-bib-0003] Bulters, D. , B. Gaastra , A. Zolnourian , et al. 2018. “Haemoglobin Scavenging in Intracranial Bleeding: Biology and Clinical Implications.” Nature Reviews Neurology 14, no. 7: 416–432. 29925923 10.1038/s41582-018-0020-0

[brb370966-bib-0004] Chen, J. , Z. Shi , C. Zhang , K. Xiong , W. Zhao , and Y. Wang . 2024. “Oroxin A Alleviates Early Brain Injury After Subarachnoid Hemorrhage by Regulating Ferroptosis and Neuroinflammation.” Journal of Neuroinflammation 21, no. 1: 116. 38702778 10.1186/s12974-024-03099-3 PMC11069275

[brb370966-bib-0005] Chen, J. , C. Zhang , T. Yan , et al. 2021. “Atorvastatin Ameliorates Early Brain Injury After Subarachnoid Hemorrhage via Inhibition of Pyroptosis and Neuroinflammation.” Journal of Cellular Physiology 236, no. 10: 6920–6931. 33792028 10.1002/jcp.30351

[brb370966-bib-0006] Chen, S. , H. Feng , P. Sherchan , et al. 2014. “Controversies and Evolving New Mechanisms in Subarachnoid Hemorrhage.” Progress in Neurobiology 115: 64–91. 24076160 10.1016/j.pneurobio.2013.09.002 PMC3961493

[brb370966-bib-0007] Chiangjong, W. , P. Netsirisawan , S. Hongeng , and S. Chutipongtanate . 2021. “Red Blood Cell Extracellular Vesicle‐Based Drug Delivery: Challenges and Opportunities.” Frontiers in Medicine 8: 761362. 35004730 10.3389/fmed.2021.761362 PMC8739511

[brb370966-bib-0008] Colombo, M. , G. Raposo , and C. Théry . 2014. “Biogenesis, Secretion, and Intercellular Interactions of Exosomes and Other Extracellular Vesicles.” Annual Review of Cell and Developmental Biology 30: 255–289. 10.1146/annurev-cellbio-101512-122326 25288114

[brb370966-bib-0009] Coulibaly, A. P. , and J. J. Provencio . 2020. “Aneurysmal Subarachnoid Hemorrhage: An Overview of Inflammation‐Induced Cellular Changes.” Neurotherapeutics 17, no. 2: 436–445. 31907877 10.1007/s13311-019-00829-x PMC7283430

[brb370966-bib-0010] Deng, X. , Y. Wu , Z. Hu , et al. 2023. “The Mechanism of Ferroptosis in Early Brain Injury After Subarachnoid Hemorrhage.” Frontiers in Immunology 14: 1191826. 37266433 10.3389/fimmu.2023.1191826 PMC10229825

[brb370966-bib-0011] Fan, H. , R. Ding , W. Liu , et al. 2021. “Heat Shock Protein 22 Modulates NRF1/TFAM‐Dependent Mitochondrial Biogenesis and DRP1‐Sparked Mitochondrial Apoptosis Through AMPK‐PGC1α Signaling Pathway to Alleviate the Early Brain Injury of Subarachnoid Hemorrhage in Rats.” Redox Biology 40: 101856. 33472123 10.1016/j.redox.2021.101856 PMC7816003

[brb370966-bib-0012] Galleggiante, V. , S. De Santis , M. Liso , et al. 2019. “Quercetin‐Induced miR‐369‐3p Suppresses Chronic Inflammatory Response Targeting C/EBP‐β.” Molecular Nutrition & Food Research 63, no. 19: e1801390. 31338984 10.1002/mnfr.201801390

[brb370966-bib-0013] Gaschler, M. M. , A. A. Andia , H. Liu , et al. 2018. “FINO2 Initiates Ferroptosis Through GPX4 Inactivation and Iron Oxidation.” Nature Chemical Biology 14, no. 5: 507–515. 29610484 10.1038/s41589-018-0031-6 PMC5899674

[brb370966-bib-0014] Ge, S. , Z. Jing , L. Wang , X. Cui , X. Zhang , and X. Wang . 2024. “Iron Metabolism and Ferroptosis in Early Brain Injury After Subarachnoid Haemorrhage.” Molecular Neurobiology 61, no. 12: 10736–10746. 10.1007/s12035-024-04218-0IF:4.6Q1. 38777982 PMC11584420

[brb370966-bib-0015] Haroon, K. , H. Zheng , S. Wu , et al. 2024. “Engineered Exosomes Mediated Targeted Delivery of Neuroprotective Peptide NR2B9c for the Treatment of Traumatic Brain Injury.” International Journal of Pharmaceutics 649: 123656. 38040392 10.1016/j.ijpharm.2023.123656

[brb370966-bib-0016] Hartl, N. , B. Gabold , F. Adams , et al. 2023. “Overcoming the Blood‐Brain Barrier?—Prediction of Blood‐Brain Permeability of Hydrophobically Modified Polyethylenimine Polyplexes for siRNA Delivery Into the Brain With In Vitro and In Vivo Models.” Journal of Controlled Release 360: 613–629. 37437848 10.1016/j.jconrel.2023.07.019

[brb370966-bib-0017] Kapralov, A. A. , Q. Yang , H. H. Dar , et al. 2020. “Redox Lipid Reprogramming Commands Susceptibility of Macrophages and Microglia to Ferroptotic Death.” Nature Chemical Biology 16, no. 3: 278–290. 32080625 10.1038/s41589-019-0462-8 PMC7233108

[brb370966-bib-0018] Khongkow, M. , T. Yata , S. Boonrungsiman , U. R. Ruktanonchai , D. Graham , and K. Namdee . 2019. “Surface Modification of Gold Nanoparticles With Neuron‐Targeted Exosome for Enhanced Blood‐Brain Barrier Penetration.” Scientific Reports 9, no. 1: 8278. 31164665 10.1038/s41598-019-44569-6 PMC6547645

[brb370966-bib-0019] Krishnan, M. A. , O. A. Alimi , T. Pan , et al. 2024. “Engineering Neurotoxin‐Functionalized Exosomes for Targeted Delivery to the Peripheral Nervous System.” Pharmaceutics 16, no. 1: 102. 38258111 10.3390/pharmaceutics16010102 PMC10818718

[brb370966-bib-0020] Krylova, S. V. , and D. Feng . 2023. “The Machinery of Exosomes: Biogenesis, Release, and Uptake.” International Journal of Molecular Sciences 24, no. 2: 1337. 36674857 10.3390/ijms24021337 PMC9865891

[brb370966-bib-0021] Kusaka, G. , M. Ishikawa , A. Nanda , D. N. Granger , and J. H. Zhang . 2004. “Signaling Pathways for Early Brain Injury After Subarachnoid Hemorrhage.” Journal of Cerebral Blood Flow and Metabolism 24, no. 8: 916–925. 15362722 10.1097/01.WCB.0000125886.48838.7E

[brb370966-bib-0022] Lai, N. , D. Wu , T. Liang , et al. 2020. “Systemic Exosomal miR‐193b‐3p Delivery Attenuates Neuroinflammation in Early Brain Injury After Subarachnoid Hemorrhage in Mice.” Journal of Neuroinflammation 17, no. 1: 74. 32098619 10.1186/s12974-020-01745-0 PMC7041199

[brb370966-bib-0023] Lauzier, D. C. , K. Jayaraman , J. Y. Yuan , et al. 2023. “Early Brain Injury After Subarachnoid Hemorrhage: Incidence and Mechanisms.” Stroke: A Journal of Cerebral Circulation 54, no. 5: 1426–1440. 10.1161/STROKEAHA.122.040072 PMC10243167 36866673

[brb370966-bib-0024] Liu, M. , K. Jayaraman , A. J. Norris , et al. 2023. “Isoflurane Conditioning‐Induced Delayed Cerebral Ischemia Protection in Subarachnoid Hemorrhage‐Role of Inducible Nitric Oxide Synthase.” Journal of the American Heart Association 12, no. 14: e029975. 37449587 10.1161/JAHA.123.029975 PMC10382105

[brb370966-bib-0025] Liu, P. , C. Ma , Q. Wu , et al. 2019. “MiR‐369‐3p Participates in Endometrioid Adenocarcinoma via the Regulation of Autophagy.” Cancer Cell International 19: 178. 31337985 10.1186/s12935-019-0897-8 PMC6624956

[brb370966-bib-0026] Mehta, A. , and D. Baltimore . 2016. “MicroRNAs as Regulatory Elements in Immune System Logic.” Nature Reviews Immunology 16, no. 5: 279–294. 10.1038/nri.2016.40 27121651

[brb370966-bib-0027] Mittelbrunn, M. , C. Gutiérrez‐Vázquez , C. Villarroya‐Beltri , et al. 2011. “Unidirectional Transfer of MicroRNA‐Loaded Exosomes From T Cells to Antigen‐Presenting Cells.” Nature Communications 2: 282. 10.1038/ncomms1285 PMC3104548 21505438

[brb370966-bib-0028] O'Connell, R. M. , D. S. Rao , A. A. Chaudhuri , and D. Baltimore . 2010. “Physiological and Pathological Roles for MicroRNAs in the Immune System.” Nature Reviews Immunology 10, no. 2: 111–122. 10.1038/nri2708 20098459

[brb370966-bib-0029] Pardridge, W. M. 2009. “Alzheimer's Disease Drug Development and the Problem of the Blood‐Brain Barrier.” Alzheimer's & Dementia 5, no. 5: 427–432. 10.1016/j.jalz.2009.06.003 PMC2756824 19751922

[brb370966-bib-0030] Peng, B. , Y. Yang , Z. Wu , et al. 2023. “Red Blood Cell Extracellular Vesicles Deliver Therapeutic siRNAs to Skeletal Muscles for Treatment of Cancer Cachexia.” Molecular Therapy 31, no. 5: 1418–1436. 37016578 10.1016/j.ymthe.2023.03.036 PMC10188904

[brb370966-bib-0031] Qu, M. , Q. Lin , L. Huang , et al. 2018. “Dopamine‐Loaded Blood Exosomes Targeted to Brain for Better Treatment of Parkinson's Disease.” Journal of Controlled Release 287: 156–166. 30165139 10.1016/j.jconrel.2018.08.035

[brb370966-bib-0032] Qu, W. , Y. Cheng , W. Peng , et al. 2022. “Targeting iNOS Alleviates Early Brain Injury After Experimental Subarachnoid Hemorrhage via Promoting Ferroptosis of M1 Microglia and Reducing Neuroinflammation.” Molecular Neurobiology 59, no. 5: 3124–3139. 35262869 10.1007/s12035-022-02788-5

[brb370966-bib-0033] Ransohoff, R. M. , and A. E. Cardona . 2010. “The Myeloid Cells of the Central Nervous System Parenchyma.” Nature 468, no. 7321: 253–262. 21068834 10.1038/nature09615

[brb370966-bib-0034] Rui, T. , H. Wang , Q. Li , et al. 2021. “Deletion of Ferritin H in Neurons Counteracts the Protective Effect of Melatonin Against Traumatic Brain Injury‐Induced Ferroptosis.” Journal of Pineal Research 70, no. 2: e12704. 33206394 10.1111/jpi.12704

[brb370966-bib-0035] Scalavino, V. , M. Liso , E. Cavalcanti , et al. 2020. “miR‐369‐3p Modulates Inducible Nitric Oxide Synthase and Is Involved in Regulation of Chronic Inflammatory Response.” Scientific Reports 10, no. 1: 15942. 32994523 10.1038/s41598-020-72991-8 PMC7525504

[brb370966-bib-0036] Scalavino, V. , E. Piccinno , A. M. Valentini , et al. 2023. “miR‐369‐3p Modulates Intestinal Inflammatory Response via BRCC3/NLRP3 Inflammasome Axis.” Cells 12, no. 17: 2184. 37681916 10.3390/cells12172184 PMC10486421

[brb370966-bib-0037] Shi, Y. , D. Yan , C. Nan , et al. 2024. “Salvianolic Acid A Inhibits Ferroptosis and Protects Against Intracerebral Hemorrhage.” Scientific Reports 14, no. 1: 12427. 38816543 10.1038/s41598-024-63277-4 PMC11140002

[brb370966-bib-0038] Singh, R. P. , I. Massachi , S. Manickavel , et al. 2013. “The Role of miRNA in Inflammation and Autoimmunity.” Autoimmunity Reviews 12, no. 12: 1160–1165. 23860189 10.1016/j.autrev.2013.07.003

[brb370966-bib-0039] Tang, D. , X. Chen , R. Kang , and G. Kroemer . 2021. “Ferroptosis: Molecular Mechanisms and Health Implications.” Cell Research 31, no. 2: 107–125. 33268902 10.1038/s41422-020-00441-1 PMC8026611

[brb370966-bib-0040] Tang, Z. , S. Hu , Z. Wu , and M. He . 2023. “Therapeutic Effects of Engineered Exosome‐Based miR‐25 and miR‐181a Treatment in Spinocerebellar Ataxia Type 3 Mice by Silencing ATXN3.” Molecular Medicine 29, no. 1: 96. 37438701 10.1186/s10020-023-00695-6 PMC10337053

[brb370966-bib-0041] Tao, W. , G. Zhang , C. Liu , L. Jin , X. Li , and S. Yang . 2023. “Low‐Dose LPS Alleviates Early Brain Injury After SAH by Modulating Microglial M1/M2 Polarization via USP19/FOXO1/IL‐10/IL‐10R1 Signaling.” Redox Biology 66: 102863. 37672892 10.1016/j.redox.2023.102863 PMC10494318

[brb370966-bib-0042] Terstappen, G. C. , A. H. Meyer , R. D. Bell , and W. Zhang . 2021. “Strategies for Delivering Therapeutics Across the Blood‐Brain Barrier.” Nature Reviews Drug Discovery 20, no. 5: 362–383. 33649582 10.1038/s41573-021-00139-y

[brb370966-bib-0043] Tian, T. , H. X. Zhang , C. P. He , et al. 2018. “Surface Functionalized Exosomes as Targeted Drug Delivery Vehicles for Cerebral Ischemia Therapy.” Biomaterials 150: 137–149. 29040874 10.1016/j.biomaterials.2017.10.012

[brb370966-bib-0044] Tian, Y. , B. Liu , Y. Li , et al. 2022. “Activation of RARα Receptor Attenuates Neuroinflammation After SAH via Promoting M1‐to‐M2 Phenotypic Polarization of Microglia and Regulating Mafb/Msr1/PI3K‐Akt/NF‐κB Pathway.” Frontiers in Immunology 13: 839796. 35237277 10.3389/fimmu.2022.839796 PMC8882645

[brb370966-bib-0045] Tu, W. J. , Z. Zhao , P. Yin , et al. 2023. “Estimated Burden of Stroke in China in 2020.” JAMA Network Open 6, no. 3: e231455. 36862407 10.1001/jamanetworkopen.2023.1455 PMC9982699

[brb370966-bib-0046] Usman, W. M. , T. C. Pham , Y. Y. Kwok , et al. 2018. “Efficient RNA Drug Delivery Using Red Blood Cell Extracellular Vesicles.” Nature Communications 9: 2359. 10.1038/s41467-018-04791-8 PMC6004015 29907766

[brb370966-bib-0047] Wang, J. , W. Tang , M. Yang , et al. 2021. “Inflammatory Tumor Microenvironment Responsive Neutrophil Exosomes‐Based Drug Delivery System for Targeted Glioma Therapy.” Biomaterials 273: 120784. 33848731 10.1016/j.biomaterials.2021.120784

[brb370966-bib-0048] Wei, Y. , J. Chen , G. E. Cai , et al. 2021. “Rosmarinic Acid Regulates Microglial M1/M2 Polarization via the PDPK1/Akt/HIF Pathway Under Conditions of Neuroinflammation.” Inflammation 44, no. 1: 129–147. 32940818 10.1007/s10753-020-01314-w

[brb370966-bib-0049] Wortzel, I. , S. Dror , C. M. Kenific , and D. Lyden . 2019. “Exosome‐Mediated Metastasis: Communication From a Distance.” Developmental Cell 49, no. 3: 347–360. 31063754 10.1016/j.devcel.2019.04.011

[brb370966-bib-0050] Yang, T. , N. Zhang , Y. Liu , et al. 2024. “Nanoplatelets Modified With RVG for Targeted Delivery of miR‐375 and Temozolomide to Enhance Gliomas Therapy.” Journal of Nanobiotechnology 22, no. 1: 623. 39402578 10.1186/s12951-024-02895-6 PMC11476726

[brb370966-bib-0051] Zheng, B. , T. Zheng , L. Wang , X. Chen , C. Shi , and S. Zhao . 2010. “Aminoguanidine Inhibition of iNOS Activity Ameliorates Cerebral Vasospasm After Subarachnoid Hemorrhage in Rabbits via Restoration of Dysfunctional Endothelial Cells.” Journal of the Neurological Sciences 295, no. 1‐2: 97–103. 20537662 10.1016/j.jns.2010.04.012

[brb370966-bib-0052] Zheng, M. , M. Huang , X. Ma , H. Chen , and X. Gao . 2019. “Harnessing Exosomes for the Development of Brain Drug Delivery Systems.” Bioconjugate Chemistry 30, no. 4: 994–1005. 30855944 10.1021/acs.bioconjchem.9b00085

[brb370966-bib-0053] Zheng, Z. V. , H. Lyu , S. Y. E. Lam , P. K. Lam , W. S. Poon , and G. K. C. Wong . 2020. “The Dynamics of Microglial Polarization Reveal the Resident Neuroinflammatory Responses After Subarachnoid Hemorrhage.” Translational Stroke Research 11, no. 3: 433–449. 31628642 10.1007/s12975-019-00728-5

